# Combining pharmacological and non-invasive brain stimulation strategies to promote remyelination in multiple sclerosis

**DOI:** 10.3389/fnmol.2026.1980126

**Published:** 2026-10-14

**Authors:** Thomas Jonathan Scheinok, Dimitri De Bundel, Tim Vanmierlo, Jeroen Van Schependom

**Affiliations:** 1 AIMS Lab, Center for Neurosciences (C4N), UZ Brussel, Vrije Universiteit Brussel, Brussels, Belgium; 2 Laboratory of Pharmaceutical Chemistry, Drug Analysis and Drug Information (FASC), Research Group Experimental Pharmacology (EFAR), Center for Neurosciences (C4N), Vrije Universiteit Brussel (VUB), Brussels, Belgium; 3 NEUR Research Group, Center for Neurosciences (C4N), Vrije Universiteit Brussel, Brussels, Belgium; 4 Division of Translational Neuroscience, Department of Psychiatry and Neuropsychology, Mental Health and Neuroscience Research Institute (MHeNS), Maastricht University, Maastricht, Netherlands; 5 Department of Neuroscience, Biomedical Research Institute (BIOMED), Hasselt University, Diepenbeek, Belgium; 6 Department of Electronics and Informatics (ETRO), Vrije Universiteit Brussel, Brussels, Belgium

**Keywords:** combination therapy, microglia, multiple sclerosis, neuroinflammation, non-invasive brain stimulation, oligodendrocyte precursor cells, remyelination

## Abstract

Remyelination in multiple sclerosis (MS) fails for several, partly overlapping reasons. The differentiation of oligodendrocyte precursor cells into myelin-producing oligodendrocytes is impaired both by intrinsic factors and by the surrounding lesion environment, which becomes increasingly inflammatory and senescent with age. Pharmacological strategies acting on a single target to promote oligodendrocyte lineage progression have been successful in animal models but have yet to produce meaningful clinical benefit in people with MS (pwMS). In this perspective paper, we argue that simultaneously targeting several of the mechanisms underlying this failure is a more promising route to repairing myelin lesions in pwMS. We outline how non-invasive brain stimulation (NIBS) might be leveraged to shift the glial microenvironment towards a pro-regenerative, remyelinating state, and how this can be paired with targeted pharmacological agents that enhance oligodendrocyte differentiation. We also discuss how the expected effect of such a combination may depend on age and lesion stage. This framework may offer a rational route beyond the translational failures of single-target remyelination therapies.

## 1 Introduction

Multiple sclerosis (MS) is a chronic, disabling disease of the central nervous system characterized by immune-mediated demyelination, incomplete remyelination, and progressive neuronal degeneration. It is believed that an initial infection with the Epstein-Barr virus dysregulates the innate immune system, resulting in autoreactive T and B cells targeting myelin antigens via a mechanism called molecular mimicry, whereby epitopes on the viral protein EBNA1 cross-react with myelin antigens, triggering an autoimmune cascade (Behrens et al., 2024). These peripheral lymphocytes breach the blood-brain barrier, triggering an immune response against myelin antigens and leading to a demyelinating insult. Clinically, such an insult might manifest as subacute focal neurological symptoms (Bjornevik et al., 2023). Early in the disease course of MS, endogenous remyelination by oligodendrocyte (OL) precursor cells (OPCs) and, to a lesser extent, surviving OLs often restores the myelin sheath to near-complete remyelination. However, with ageing, this repair mechanism deteriorates, and remyelination becomes aberrant, leaving denuded axons vulnerable to injury, axonal death, and neurological disability (Neumann et al., 2019b).

Several compounds that enhance OL lineage progression in experimental systems have advanced to clinical testing, but none has so far produced a reproducible improvement in disability that can be attributed to remyelination (Mei et al., 2014; Mi et al., 2007). For instance, clemastine met its primary electrophysiological endpoint in the ReBUILD trial, shortening visual evoked potential (VEP) P100 latency by 1.7 ms per eye, and subsequent myelin water imaging provided convergent evidence of increased myelin content in the corpus callosum (Caverzasi et al., 2023; Green et al., 2017). Nevertheless, the magnitude and clinical relevance of this latency change remain uncertain, and a clemastine study in progressive MS was halted after an unexpected acceleration of disability (Kocot et al., 2025). One possible explanation is that many of these approaches primarily target oligodendrocyte lineage progression, while insufficiently addressing the non-permissive inflammatory, senescent, and extracellular-matrix-rich microenvironment of MS lesions (Allanach et al., 2022). Importantly, a recent study using the experimental autoimmune encephalomyelitis (EAE) mouse model demonstrated that combining an anti-CD20 compound with clemastine fumarate significantly enhanced remyelination, reduced inflammatory activity, and improved functional recovery compared to either treatment alone (Li et al., 2025). Similar results were obtained with a different combinatorial approach using tuftsin, an immunomodulatory tetrapeptide that induces an anti-inflammatory shift in microglia and macrophages, combined with benztropine, an anticholinergic drug with remyelinating capacity (Thompson et al., 2018).

Non-invasive brain stimulation (NIBS) refers to techniques that modulate neural network activity without the necessity for surgery, consisting of repetitive transcranial magnetic stimulation (rTMS), transcranial direct current stimulation (tDCS), and transcranial alternating current stimulation (tACS). By altering neuronal firing, oscillatory synchronization, and neuron–glia signalling, NIBS may engage distinct repair mechanisms (Scheinok et al., 2023). In this perspective paper, we argue that remyelination failure in MS should not be viewed solely as a failure of oligodendrocyte precursor cell differentiation, but also as a consequence of a non-permissive inflammatory and age-related glial microenvironment. We therefore propose that future pro-remyelinating strategies should target both intrinsic oligodendrocyte lineage progression and the extrinsic lesion environment in which remyelination occurs. In this context, non-invasive brain stimulation may represent a complementary, activity- and neuromodulator-dependent approach to modulating neuron–glia signalling and shifting the glial microenvironment towards a more pro-regenerative state. We therefore outline the mechanistic rationale for combining NIBS with pharmacological strategies that either enhance oligodendrocyte differentiation or remodel the inflammatory lesion microenvironment, while highlighting the need to avoid mechanistic overlap with neurotransmitter-receptor pathways involved in NIBS-induced glial modulation. We also discuss how ageing of the axon-myelin unit and lesion stage may determine whether such a combination can be expected to work.

## 2 Understanding the role of the inflammatory micro-environment in remyelination

Successful remyelination depends on different sequential phases: myelin debris clearance, recruitment of glial cells to the demyelinated area, OPC differentiation, and myelin production. Failure at any of these stages results in incomplete remyelination and subsequent axonal degeneration (Neumann et al., 2019b).

Following a demyelinating insult, microglia are rapidly activated and shift towards a pro-inflammatory transcriptional state, characterized by the expression of inducible nitric oxide synthase (iNOS), tumour necrosis factor-α (TNF-α), and various other chemokines. This activation is a prerequisite for successful remyelination as it is necessary for the initiation of myelin debris clearance and recruitment of OLs to the lesion site. Interestingly, remyelination requires the necroptosis of microglia in a pro-inflammatory transcriptional state, whereafter a new pool of microglia exhibiting a pro-regenerative transcriptional state can populate the lesion site (Lloyd et al., 2019).

Pro-regenerative microglia express various signalling molecules that activate OPCs and drive their differentiation, including Arginase-1, CD206, and insulin-like growth factor-1 (IGF-1) (Miron et al., 2013). However, with ageing, this process becomes inefficient as microglia remain in an inflammatory state. Microglia that accumulate in demyelinated lesions of aged animals express a senescence-associated secretory phenotype (SASP), characterized by chronic upregulation of pro-inflammatory mediators and impaired phagocytic activity. As these microglia do not transition into pro-regenerative transcriptional states, remyelination largely fails. Specifically, the expression of several factors, such as CD36, is decreased, thereby impairing the phagocytic activity of microglia and reducing myelin debris clearance (Dema et al., 2021). This clearance is crucial because myelin debris contains several signalling molecules that inhibit OL differentiation, such as myelin-associated glycoprotein (MAG) and Neurite Outgrowth Inhibitor-A (NOGO-A) (Hammel et al., 2022). Simultaneously, pro-inflammatory cytokines such as IL-1β and TNF-α, along with reactive oxygen species, are released, creating a hostile microenvironment for OL development (Dema et al., 2021). Parabiosis experiments have shown that blood from young mice can restore remyelinating capacity in aged mice by recruiting pro-regenerative microglia, indicating that rejuvenating microglial phenotypes may be a potential target for remyelination in pwMS (Dema et al., 2021).

Like microglia, astrocytes can exhibit different transcriptional states that differentially impact MS. In the early phases of remyelination, pro-inflammatory phenotypes are expressed in the active lesion (Rawji et al., 2020). This activation is mediated through the secretion of pro-inflammatory factors by microglia, such as TNF-α, IL-1β, and C1q, which induce astrocyte reactivity. Reactive astrocytes phagocytose myelin debris and, in turn, secrete chemokines, enabling the recruitment of microglia and peripheral immune cells (Liddelow et al., 2017). Through a complex molecular reprogramming mechanism, astrocytes adopt a pro-regenerative phenotype, aiding oligodendrogenesis and remyelination. Pathways such as the cholesterol biosynthesis pathway are essential for this purpose (Molina-Gonzalez et al., 2023). However, with ageing, this transition becomes impaired; the production of growth factors supporting OL survival decreases, and reactive astrocytes secrete extracellular matrix (ECM) proteins, thereby producing a glial scar and neurotoxins, further contributing to remyelination failure (Palmer and Ousman, 2018).

Ageing of white matter is, however, not restricted to glial senescence. The axon-myelin unit itself is subjected to age-related changes. In the ageing brain, myelin progressively degenerates, and the myelin debris accumulates in the microglia lysosomes. This increases the clearance burden on ageing microglia that are already less efficient (Safaiyan et al., 2016). Myelinated axons also require energy supply from OLs as OLs provide glycolytic products such as glucose and lactate to maintain axonal integrity (Fünfschilling et al., 2012). This support is reduced in demyelinated or thinly remyelinated axons while the energy cost of conduction increases (Campbell and Mahad, 2011). Furthermore, ageing white matter is more vulnerable to metabolic and ischemic stress as demonstrated by Baltan et al. who demonstrated that optic nerves from older mice were more severely injured by oxygen-glucose deprivation than those of young mice (Baltan et al., 2008). This may be relevant in older pwMS in which vascular comorbidities accumulate.

Several preclinical studies have successfully improved the glial microenvironment and promoted oligodendrogenesis and remyelination. For instance, molecules targeting glial inflammatory signalling, such as PPARγ agonists and IL-4, were found to enhance OL development and remyelination (Zhang et al., 2019). Similarly, Natarajan et al. (2016) demonstrated that the Toll-like receptor 3 agonist Poly-IC increased interleukin-33 production in oligodendrocyte precursor cells, thereby stimulating myelin gene transcription and cell maturation. Using Poly-IC in a demyelinating mouse model led to increased OPC recruitment and a shift in the glial microenvironment toward a pro-regenerative state. Likewise, remodeling the ECM with inhibitors of chondroitin sulfate proteoglycan synthesis led to enhanced recruitment and differentiation of OPCs, followed by subsequent remyelination (Keough et al., 2016). Therefore, manipulating the glial microenvironment towards a state conducive to remyelination appears to be a prerequisite for pharmacological approaches to be effective.

## 3 Effects of neuronal activity on glial cells and remyelination

A mounting body of evidence from healthy and demyelinating animal models suggests that modulating neuronal activity can positively affect OL proliferation, differentiation, and maturation, as well as subsequent remyelination (Scheinok et al., 2023). Direct synaptic communication between axons and OPCs is mediated through different neurotransmitters for which receptors are expressed on OPCs. Optogenetic stimulation of a subset of neurons demonstrates the neuronal activity-dependent maturation of OLs, while blocking synaptic release impairs CNS myelination (Gibson et al., 2014; Mensch et al., 2015). Further evidence is provided by an ethidium bromide myelin lesion model, in which tetrodotoxin (TTX), an action potential-inhibiting toxin, impaired OL development and axon remyelination (Gautier et al., 2015). This reveals the importance of axonal signalling in remyelination.

OPCs represent a special population of glial cells that maintain their proliferative and differentiation capacities throughout life, though the efficiency of these processes varies with age and brain region (Spitzer et al., 2019). These cells express various neurotransmitter receptors, allowing synaptic communication between OPCs and neurons. Various in vitro experiments have found that silencing those receptors can disrupt the development of these cells (Habermacher et al., 2019). Conversely, stimulating these receptors is feasible by inducing electrical currents into neuron-OPC cultures and can effectively boost oligodendrogenesis (Lee et al., 2017). Interestingly, the pattern of neuronal activity seems to influence the behaviour of OPCs, with high-frequency stimulation boosting proliferation while low-frequency electrical activity promoting differentiation (Nagy et al., 2017).

Neuronal activity can be modulated through invasive methods such as optogenetics or chemogenetics but also through non-invasive brain stimulation (NIBS). The underlying mechanisms of neuronal activity-induced myelin repair are not yet fully elucidated but include neuron-OPC communication through neurotransmitters such as glutamate, GABA and purinergic molecules as well as the release of several neurotrophic factors from neurons such as brain derived neurotrophic factor (BDNF), finally driving activity-dependent (re)myelination (Zhou and Zhang, 2023).

Importantly, activity-dependent effects on remyelination are unlikely to be mediated exclusively through direct neuron–OPC communication. Astrocytes and microglia also express receptors for neurotransmitters and neuromodulators, allowing them to respond directly to extracellular signalling changes induced by neuronal activity or NIBS. For example, neuronal depolarization triggers potassium release at nodes of Ranvier, which is sensed by microglia via the potassium channel THIK-1, inducing a shift towards pro-regenerative microglial transcriptional states necessary for myelin debris clearance (Ronzano et al., 2021). In astrocytes, neurotransmitter and neuromodulator signalling can induce intracellular calcium responses that influence astrocytic phenotype and function. For instance, tDCS-induced astrocytic calcium signalling has been linked to noradrenergic alpha-1 receptor mechanisms in the healthy mouse cortex (Monai et al., 2016), illustrating that NIBS effects on glia may involve receptor-mediated neuromodulatory pathways in addition to changes in neuronal firing. Thus, NIBS may influence the lesion microenvironment through a combination of neuronal activity-dependent, neurotransmitter-mediated and neuromodulator-mediated glial pathways.

Using NIBS, it was shown that low-intensity rTMS (LI-rTMS) promotes OL survival and subsequent remyelination in an acute demyelinating mouse model (Nguyen et al., 2024). LI-rTMS delivered as intermittent theta burst stimulation (iTBS) has been shown to enhance remyelination by increasing the number of myelin internodes elaborated by new OLs and by supporting the myelinating capacity of surviving OLs (Nguyen et al., 2024). Similarly, we showed that 10 Hz tACS enhanced oligodendrogenesis and myelin repair following cuprizone-induced demyelination (Scheinok et al., 2026). These findings strengthen the rationale for NIBS as a pro-remyelinating intervention and should be clinically researched in conjunction with anti-inflammatory pharmacological agents.

Evidence for NIBS-induced glial modulation, in contrast, comes largely from models other than demyelination. For instance, rTMS at 10 Hz shows a remarkable capacity to inhibit astrocyte polarization towards a pro-inflammatory phenotype while promoting a shift towards pro-regenerative states in an ischemic rat model (Hong et al., 2020). Likewise, two-photon calcium imaging showed that tDCS can directly increase calcium signalling in astrocytes of healthy mice (Monai et al., 2016). Moreover, high-frequency rTMS can shift the microglial phenotype towards regeneration via the JAK2/STAT3 pathway, attenuating white matter lesions in a post-stroke rat model (Chen et al., 2023). A systematic review of rTMS effects on glial cells confirms that, across multiple disease models, high-frequency rTMS consistently decreases markers of astrocyte reactivity (GFAP) and attenuates microglial pro-inflammatory activation (Ferreira et al., 2024). Importantly, these glial modulatory effects appear to be context-dependent and are more pronounced in injured than in healthy brain tissue, suggesting that the therapeutic window for NIBS-mediated glial modulation may be particularly relevant in the pathological context of MS. However, it is important to note that the injury in these models is ischemic rather than immune-mediated demyelination, and the glial states involved are not necessarily the same. Of the studies discussed in this section, only Ronzano et al. (2021), Nguyen et al. (2024) and our own work (Scheinok et al., 2026) were performed in the context of demyelination. Whether the glial mechanisms described in ischemic and post-stroke models also apply to MS lesions remains to be demonstrated.

It is important to recognize that NIBS modalities are not mechanistically equivalent. While rTMS primarily acts via electromagnetic induction and predominantly targets neurons, tDCS and tACS exert weaker, more diffuse effects with documented direct actions on astrocyte and microglial signalling (Pelletier and Cicchetti, 2014). The specific effects of each NIBS modality on remyelination and glial modulation, therefore, require further characterization. These results collectively suggest that NIBS may influence remyelination through multiple, partially overlapping mechanisms: by modulating neuronal activity, by altering neurotransmitter and neuromodulator release, and by engaging receptor-mediated signalling pathways in OPCs, astrocytes and microglia. Rather than acting solely through neurons, NIBS may therefore shape the lesion microenvironment through broader neuron–glia signalling mechanisms that can promote pro-regenerative glial states and support oligodendrogenesis. Unlike pharmacological approaches that must cross the blood-brain barrier to achieve adequate tissue concentrations, NIBS can directly influence glial cells in targeted brain regions. It remains to be tested, however, whether NIBS can influence senescent glial cells as effectively as younger reactive glial cells, thereby improving the toxic microenvironment created by ageing, as in progressive MS.

To our knowledge, the preclinical NIBS studies on remyelination discussed above were all performed in young adult animals (Nguyen et al., 2024; Scheinok et al., 2026). It is therefore unknown whether similar effects can be expected in aged white matter.

## 4 Which mechanisms are expected to be complementary?

Pharmacological agents and NIBS may influence different processes of the remyelination process. Most investigated remyelinating pharmaceuticals act intrinsically on OPCs. For example, clemastine and PIPE-307 target the muscarinic M1 receptor-mediated inhibition of OPC differentiation (Mei et al., 2014; Poon et al., 2024), whereas thyromimetics and bexarotene activate myelin gene expression (Huang et al., 2011; Chaudhary et al., 2021). Metformin, on the other hand, seems to restore the responsiveness of aged OPCs to differentiation cues (Neumann et al., 2019a). In contrast, NIBS is thought to act differently and mainly seem to influence the environment in which OPCs must differentiate as well as on axonal signals that guide myelination (Scheinok et al., 2023; Ferreira et al., 2024).

Distinct complementary processes may be targeted in a combinatorial approach of NIBS and pharmacological agent. First, NIBS may shift the intrinsic states of microglia and astrocytes towards a regenerative approach and support myelin debris clearance (Ronzano et al., 2021). Second, neuronal activity influences which axons get myelinated an how many internodes are formed by (new) OLs (Gibson et al., 2014; Mensch et al., 2015; Nguyen et al., 2024). A drug that increases differentiation of OPCs may therefore benefit from a supplementary activity-dependent signal that directs these cells towards generation of myelin sheaths. Third, as discussed before, metformin may intrinsically act on the metabolism of aged OPCs, whereas NIBS could improve the toxic extrinsic glial micro-environment. Such combinatorial approach of anti-inflammatory and pro-differentiation agent has already been shown to outperform either treatment alone in a preclinical study using anti-CD20 and clemastine (Li et al., 2025).

However, some mechanisms of both approaches may counteract each other. Clemastine is thought to promote OPC differentiation mainly by antagonising muscarinic M1 receptors (Mei et al., 2014). If NIBS increases cholinergic signalling onto OPCs, activation of these same receptors might act as a brake on differentiation and partly offset the effect of clemastine. To this date, this interaction has not been investigated. It is also unlikely to be the only antagonistic interaction between the two approaches, and possible interactions should therefore be tested before these strategies are combined in pwMS.

## 5 Lesion stage and glial state

The effects of a combined approach may also depend on the lesion stage. MS lesions are classified as active, mixed active/inactive and inactive lesions (Kuhlmann et al., 2017). Failure of differentiation has distinct causes across on the state of the lesions: in active lesions, mature OLs are present but fail to form myelin sheaths; whereas in mixed active/inactive lesions OLs are depleted and a hostile micro-environment is present (Heß et al., 2020). At the edge of chronic active lesions, microglia and astrocytes remain in a persistent inflammatory state, partly driven by complement component C1q (Absinta et al., 2021).

In active inflammatory lesions, the priority is to limit ongoing injury which is mainly achieved with current disease-modifying therapies. The early pro-inflammatory glial response however is essential to clear myelin debris and a NIBS-induced shift of glial cells towards pro-regenerative states may therefore need to be delayed (Kotter et al., 2006; Lloyd et al., 2019). On the other hand, in actively remyelinating lesions the therapeutic window of NIBS combined with pro-differentiation drugs seems to be the largest as OPCs and newly formed OLs are present (Franklin and Simons, 2022). In chronic active and inactive lesions with persistent glial activation or senescence, pro-differentiation drugs alone are unlikely to be effective, as both OPCs are depleted and the micro-environment in which differentiation is supposed to occur is hostile. In these lesions, the glial state would first have to be altered before a pro-differentiation drug can act. Whether NIBS is able to reverse persistent glial activation or senescence is to this date unknown. However, adding a drug such as metformin that may rejuvenate OPCs can offer an elegant solution to this problem, yet has to be investigated further in this context (Neumann et al., 2019a). Future studies with combined approaches should also stratify outcomes by lesion type, for instance by using paramagnetic rim lesions on MRI as a marker of chronic active lesions.

## 6 Future perspectives

The translational failures of remyelination pharmacotherapy provide valuable lessons for future therapies. Several pharmacological agents (summarized in Table 1) have yielded promising results in preclinical models but have not shown similar effects in pwMS. For instance, clemastine fumarate, an antihistaminic drug with antimuscarinic properties, was shown to promote the differentiation of OLs preclinically but only modestly enhanced myelin repair (e.g., decreased visual evoked potential latency times) in ReBUILD trial. Moreover, higher doses may be effective but entail serious side effects, limiting their use in daily practice (Kocot et al., 2025). Alternatively, using PIPE-307, a more selective M1 receptor antagonist, may address the unfavourable side-effect profile. This agent was shown to enhance remyelination in preclinical studies, including the EAE model, and was evaluated in the phase 2 VISTA trial (NCT06083753) of relapsing-remitting MS (Poon et al., 2024). The VISTA trial has recently reported results: while PIPE-307 was well-tolerated, it failed to meet its primary endpoint of improvement in low-contrast letter acuity, illustrating the translational challenges of bringing pro-remyelinating strategies from preclinical to clinical research. Another drug enhancing OL differentiation in preclinical trials, anti-LINGO 1 (opicinumab), was not found to be effective in delaying disease progression in pwMS (Gharagozloo et al., 2022).

**Table 1:** Representative pharmacological strategies relevant to combination with NIBS.

Intervention / target	Proposed repair mechanism	Current evidence
Clemastine; broad H1/M1 antimuscarinic activity	Promotes OPC differentiation, most likely through M1 receptor antagonism (Mei et al., 2014).	ReBUILD showed a small but significant improvement in VEP latency (Green et al., 2017), with later myelin water fraction evidence of remyelination (Caverzasi et al., 2023); fatigue was common. A progressive-MS study raised a safety signal for accelerated disability (Kocot et al., 2025).
PIPE-307; selective M1 antagonist	Retains pro-differentiation activity while reducing off-target anticholinergic effects.	Promoted remyelination in preclinical models (Poon et al., 2024). The phase II VISTA trial in relapsing-remitting MS reported acceptable safety at both doses but did not meet its primary or secondary efficacy endpoints (Contineum Therapeutics, press release, 20 November 2025).
Metformin; AMPK/metabolic pathways	Rejuvenates aged OPCs and alters mitochondrial metabolism, restoring their responsiveness to differentiation cues.	Improved remyelination in aged rodents (Neumann et al., 2019a) and enhanced human oligodendrocyte function in cell-based systems (Kazakou et al., 2025); clinical add-on studies include CCMR Two (Riboni-Verri et al., 2025) and MACSiMiSE-BRAIN (De Keersmaecker et al., 2024).
Liothyronine and CNS-selective thyromimetics	Thyroid-hormone-receptor signalling promotes oligodendrocyte differentiation, lipid synthesis and myelin production.	Liothyronine was well tolerated in a phase I dose-ranging study but without proof of efficacy (Wooliscroft et al., 2020); sobetirome/Sob-AM2 and newer thyromimetics are supported mainly by preclinical data (Chaudhary et al., 2021; Saponaro et al., 2020).
Opicinumab; anti-LINGO-1	Removes an inhibitory signal on oligodendrocyte differentiation and axonal regeneration (Mi et al., 2007).	SYNERGY (Cadavid et al., 2019) and AFFINITY (Calabresi et al., 2026) failed to meet their primary endpoints, despite being used as add-on therapy to anti-inflammatory DMTs.
Bexarotene; retinoid X receptor agonism	Activates RXR-dependent differentiation and myelin-gene programmes.	CCMR One had a negative primary MRI endpoint and poor tolerability, with only exploratory electrophysiological and regional MRI signals (Brown et al., 2021).
Approved DMTs	Suppress new inflammatory injury; some also exert direct effects on oligodendrocytes or other glia in experimental systems.	A 2025 systematic review found suggestive imaging and electrophysiological signals for several DMTs, but insufficient evidence that these changes reflect remyelination alone (Makhzoum et al., 2025; Zuroff et al., 2025).
GPR17 antagonism	Facilitates the transition from immature OPC states towards terminal differentiation (Lewash et al., 2025)	GPR17 remains an early-development target. PTD802 is the first GPR17 antagonist to enter clinical development, with UK clinical trial authorisation and FDA IND clearance for first-in-human safety studies; no efficacy trial for remyelination in MS has been reported (Pheno Therapeutics, press release, 22 June 2026).

These failures can be partially explained by the limited translational potential of the animal models studied, such as the cuprizone model. While invaluable for researchers studying the effects of remyelinating agents, these models do not include the crucial chronic inflammatory microenvironment in which OPCs must be recruited and develop to effectively remyelinate MS lesions. The cuprizone model is particularly relevant for its predictable, near-complete remyelination phase, making it an ideal platform for testing remyelinating interventions (Kipp, 2024).

NIBS has already been tested extensively in pwMS in clinical trials. A meta-analysis of 25 randomized controlled trials (19 tDCS, 6 rTMS) found that tDCS significantly reduces fatigue with a large effect size and improves cognitive function, while rTMS reduces spasticity in pwMS (Kan et al., 2022). A broader systematic review and network meta-analysis of 49 trials confirmed that NIBS is safe and well-tolerated in pwMS, with no reports of serious adverse effects or pharmacological interactions (Zhang et al., 2022). These trials establish a safety and feasibility foundation for NIBS in MS patients. However, none of these trials were designed to assess remyelination as a primary endpoint. This represents a critical research gap, as preclinical evidence supporting NIBS-induced remyelination has not yet been validated in human MS. Future trials should incorporate advanced MRI markers of myelin content (e.g., myelin water imaging, magnetization transfer ratio) and electrophysiological measures of conduction velocity as endpoints for assessing remyelination.

Importantly, no single animal model reproduces the spatial heterogeneity, chronic compartmentalized inflammation, ageing, and neuroaxonal loss that characterize human MS. Cuprizone and focal toxin models offer controlled demyelination and temporally predictable repair, but they lack a substantial adaptive immune component. EAE, by contrast, provides an inflammatory context and can reproduce chronic visual-pathway demyelination and partial remyelination; selective PDE4 inhibition studies illustrate how inflammatory and repair mechanisms can be interrogated within a single coordinated program (Schepers et al., 2023). Aged and chronic cuprizone paradigms, chronic EAE, and sequential or hybrid models should therefore be regarded as complementary platforms, each capturing a different aspect of the disease. Aged animals in particular will be needed in order to test whether NIBS remains effective in senescent white matter. For NIBS–drug combinations specifically, factorial designs that include each monotherapy, the combination, and sham controls will be needed, with the interaction between modalities tested in advance to distinguish additive, synergistic, and antagonistic effects.

VEP latency is an attractive translational endpoint because it is inexpensive, reproducible, and sensitive to conduction along a clinically relevant pathway. Experimental loss-of-function work has shown that clemastine improves VEP latency only when new myelin can form, which provides unusually strong biological validation of latency as a preclinical remyelination marker (Cordano et al., 2022). However, VEP latency is also influenced by inflammation, temperature, axonal loss, retinal dysfunction, synaptic transmission, and various technical factors, so a small improvement in latency should not be equated with clinically meaningful repair. Future NIBS trials would therefore benefit from combining VEP or motor-evoked-potential latency with complementary markers, such as myelin water fraction, magnetization transfer or quantitative susceptibility measures, optical coherence tomography, low-contrast visual acuity, and functional outcomes, since concordance across several modalities is more informative than any single surrogate (Caverzasi et al., 2023; Cordano et al., 2022; Makhzoum et al., 2025; Zuroff et al., 2025).

Collectively, current evidence suggests that remyelination failure in MS cannot be explained solely by impaired oligodendrocyte differentiation but also reflects a non-permissive inflammatory and age-related lesion microenvironment. NIBS represents a mechanistically distinct therapeutic strategy that may be capable of modulating neuron-glia communication and potentially reshaping this microenvironment, thereby complementing pharmacological approaches aimed at oligodendrocyte lineage progression. Rather than replacing remyelinating drugs, NIBS may therefore be a useful component of combinatorial strategies. However, NIBS-induced remyelination has to this date not been demonstrated in pwMS, and most evidence for NIBS-mediated glial modulation comes from non-MS models. Future research should focus on elucidating the molecular mechanisms of NIBS-induced glial modulation in demyelination models and in human systems, validating these mechanisms in translational models that better recapitulate chronic MS pathology and ageing, and developing rational combination therapies based on complementary rather than overlapping mechanisms.

## Data Availability

The original contributions presented in the study are included in the article/supplementary material, further inquiries can be directed to the corresponding author.
